# The mitochondrial paradox

**DOI:** 10.7554/eLife.59140

**Published:** 2020-06-25

**Authors:** Sophie L Penman, Rebecca L Jensen, Robyn T Kiy, Amy E Chadwick

**Affiliations:** 1 MRC Centre for Drug Safety Science, University of LiverpoolLiverpoolUnited Kingdom; 2 Department of Molecular and Clinical Pharmacology, University of LiverpoolLiverpoolUnited Kingdom

**Keywords:** toxicophore, mitochondria, cardiac liability, anti-cancer drugs, Human

## Abstract

A structural motif that is found in two cancer drugs may be responsible for their ability to tackle cancers and for the side-effects caused by the drugs.

**Related research article** Stephenson ZA, Harvey RF, Pryde KR, Mistry S, Hardy RE, Serreli R, Chung I, Allen TE, Stoneley M, MacFarlane M, Fischer PM, Hirst J, Kellam B, Willis AE. 2020. Identification of a novel toxicophore in anti-cancer chemotherapeutics that targets mitochondrial respiratory complex I. *eLife* **9**:e55845. doi: 10.7554/eLife.55845

Organelles called mitochondria are often referred to as the powerhouse of a cell because they make the molecules of ATP that the cell uses as a source of energy. The toxic side-effects of some medicines are caused by the drug inadvertently disrupting the workings of mitochondria (Nadanaciva and Will, 2011). The heart is particularly susceptible to such side-effects because cardiac cells contain large numbers of mitochondria to meet the energy demands of heart tissue (Park et al., 2014; Varga et al., 2015). Understanding how this toxicity arises is important so it can be avoided when designing and developing new treatments. However, it can be diffucult to determine which part of the drug causes these toxic side-effects.

Now, in eLife, Anne Willis (University of Cambridge) and colleagues – including Zoë Stephenson as first author – report details of a chemical structure in the anti-cancer drug mubritinib, which inhibits the mitochondria of cardiac cells and causes an unintended rise in toxicity (Stephenson et al., 2020). Previous work had shown that mubritinib disrupts the phosphorylation of a protein called HER2 that is known to promote the growth of cancer cells (Nagasawa et al., 2006). However, during tests, Stephenson et al. found that increasing the concentration of mubritinib did not hinder this protein to the same degree as a drug called lapatinib, which is known to work by inhibiting HER2. This suggested that mubritinib does not directly inhibit HER2 and that another mechanism is likely to be responsible for its anti-cancer effects.

As mubritinib is known to affect pathways that are linked to cellular energy, the researchers – who are based at University of Cambridge and the University of Nottingham – decided to investigate how this drug impacted the production of ATP in cardiac cells cultured in two media: glucose and galactose. Cells cultured in galactose rely more heavily on mitochondria for ATP production than cells cultured in glucose, so are more susceptible to compounds that target this organelle (Marroquin et al., 2007). Stephenson et al. found that, following drug treatment, the cells in galactose produced less ATP and had a lower frequency of beating than the cells in glucose. This suggests that mubritinib impairs the activity of the electron transport chain which drives the synthesis of ATP (Figure 1). Further analysis revealed that mubritinib inhibits a particular structure within this chain called 'complex I'.

**Figure 1.**
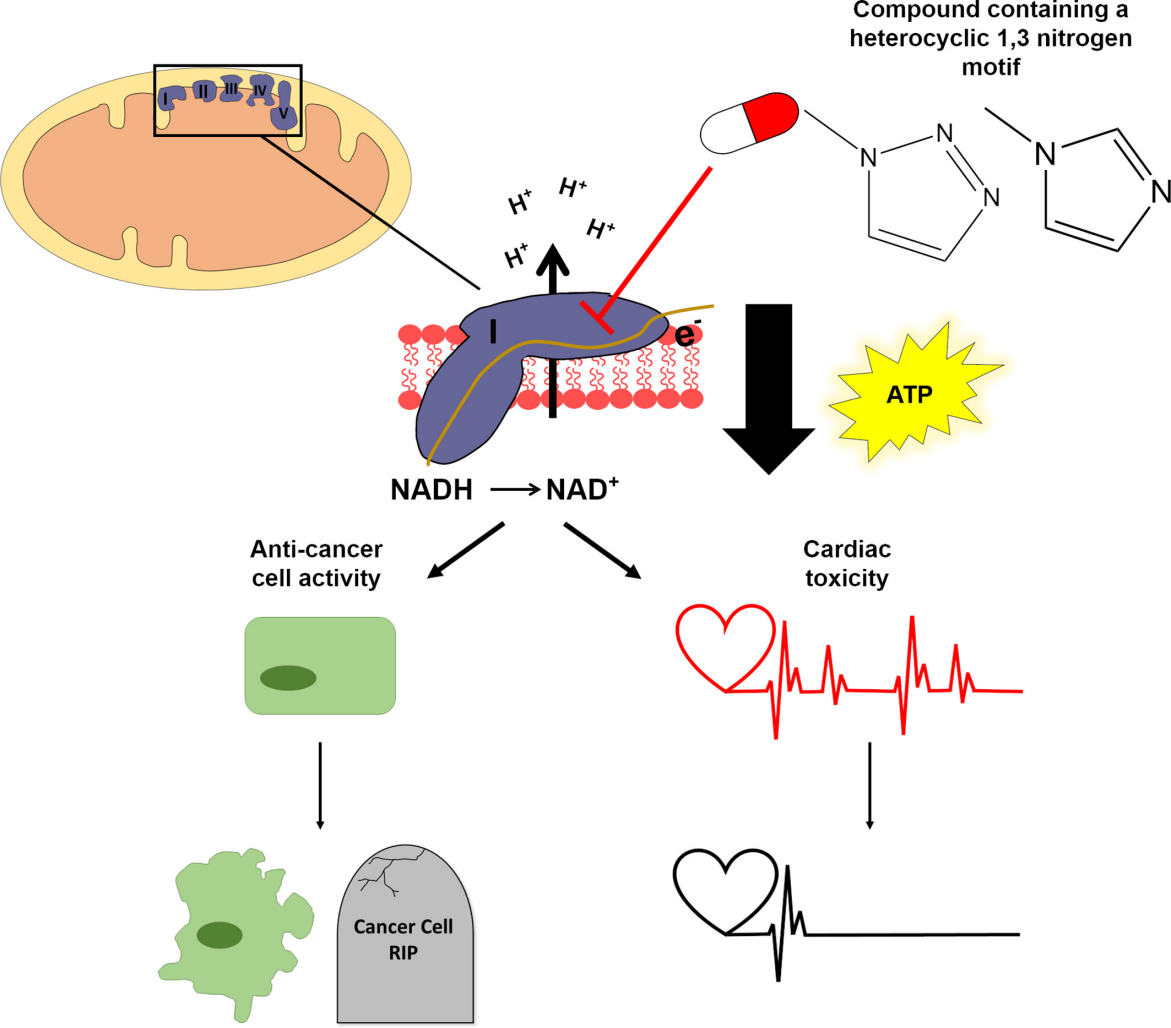
A structural motif in two anti-cancer drugs disrupts the production of ATP. Mitochondria (top left) make the ATP molecules that provide cells with energy. Chains of protein complexes called electron transporters (purple; labelled I, II, III, IV and V) are embedded in the inner membrane of mitochondria. The first complex in this chain (complex I) converts NADH to NAD+ by removing an electron (middle panel), which then gets shuttled between the different complexes in the chain. This allows the complexes to actively transport protons (H+) into the space between the inner and outer membrane of the mitochondrion. The diffusion of these protons back across the inner membrane (downward black arrow) drives the enzyme that synthesizes ATP molecules. Two anti-cancer drugs, mubritinib and carboxyamidotriazole, contain a motif (top right) which inhibits complex I and consequently disrupts the production of ATP. Stephenson et al. found that inhibiting complex I in cancer cells led to reduced growth and increased death (bottom left), whereas inhibiting complex I in cardiac cells caused the cells to beat less frequently due to the reduction in ATP (bottom right).

Image credit: Sophie Penman.

Next, Stephenson et al. investigated a library of compounds which have a similar structure to mubritinib to identify the 'toxicophore' – the region of the drug that is causing the side-effects. This revealed that a region called the heterocyclic 1,3 nitrogen motif was responsible for inhibiting complex I and reducing the production of ATP (Figure 1).

The researchers then investigated the effects of an anti-cancer drug called carboxyamidotriazole that contains the same toxicophore structure. This drug is thought to block the progress of cancer by inhibiting specific ion channels that transport calcium ions into the cell (Singh et al., 2017). However, Stephenson et al. found that carboxyamidotriazole did not significantly bind to calcium channels. Instead, they discovered that the drug reduced the production of ATP in galactose media and decreased the amount of oxygen taken up by cardiac cells.

Finally, to identify whether the heterocyclic 1,3 nitrogen motif was responsible for the anti-cancer effects of both drugs, Stephenson et al. measured the growth and death rate of cancer cells following treatment. Cell lines representing five different cancer types were treated with mubritinib, carboxyamidotriazole, or structurally similar compounds which lacked the toxicophore. In each cell line they tested, the presence of the toxicophore resulted in increased levels of cell death and reduced rates of cell growth (Figure 1). This suggests that the toxicophore in these two drugs is also partially responsible for their anti-cancer effects.

These findings provide evidence of a chemical motif which increases the toxicity of cardiac cells by inadvertently targeting mitochondria. The identification of this motif could help design safer and more effective anti-cancer treatments. Furthermore, the method used in this study could be used to identify other chemical motifs which specifically disrupt the activity of mitochondria. Future research should test a larger collection of compounds containing this toxicophore to confirm whether the loss in mitochondrial activity is linked to adverse side effects. Furthermore, it is important to assess whether these effects only cause toxicity in the heart or whether other organs, such as the liver and kidney, may also be susceptible.
